# Complete remission in a patient with sinonasal squamous cell carcinoma receiving neoadjuvant tislelizumab plus chemotherapy: a case report

**DOI:** 10.3389/fimmu.2024.1414529

**Published:** 2024-07-15

**Authors:** Fang Chen, Hongzheng Zhang, Yonghe Li, Tingfeng Liang, Tao Zhang

**Affiliations:** Department of Otorhinolaryngology Head and Neck Surgery, Zhujiang Hospital, Southern Medical University, Guangzhou, China

**Keywords:** sinonasal squamous cell carcinoma, tislelizumab, immune checkpoint inhibitor, immunotherapy, pathological complete response

## Abstract

Sinonasal squamous cell carcinoma (SNSCC) is the most common, high-aggressive sinonasal malignancies that have remained relatively stable poor outcomes over the past decade. As a first-line treatment for SNSCC, surgery plus adjuvant radiotherapy is recommended. However, complete surgical resection may not be appropriate due to the proximity of the nasal cavity and sinuses to key structures such as orbit or intracranial. Currently, immune checkpoint inhibitors (ICIs) have been established as one of the first-line therapies for many solid tumors with unresectable stage. However, evidence on the efficacy of ICIs in sinonasal malignancy is scarce and no ICIs are approved for use in SNSCC up to day. In this report, we report a case of a 64-year-old man with SNSCC treated by multi-protocol exploration. The patient achieved pathological complete response (pCR) after receiving two cycles of Docetaxel and cisplatin combined with tislelizumab. To the best of our knowledge, this is the first case of SNSCC treated with tislelizumab that achieved pCR. This case offers real-world evidence that chemotherapy plus immunotherapy is a promising treatment for SNSCC.

## Introduction

Sinonasal squamous cell carcinoma (SNSCC) is the most common sinonasal malignancies that are locally aggressive and often present at an advanced stage (1, 2). The standard treatment for sinonasal malignancies is surgical resection plus adjuvant radio-chemotherapy. However, surgical resection often carries high morbidity and adverse functional consequences in high aggressive SNSCC, despite the fact that surgical resection with negative margins improves local control and overall survival (3). Therefore, there is a need for multimodal treatment strategies to reduce significant surgical morbidity and adverse outcomes. Currently, immune checkpoint inhibitors (ICIs) have been established as one of the first-line therapies for many solid tumors with unresectable stage. However, evidence on the efficacy of ICIs in SNSCC is scarce. In this report, we present a case of advanced SNSCC who achieved pathological complete response (pCR) after two cycles of neoadjuvant treatment with immunotherapy tislelizumab in combination with DP regimen chemotherapy (docetaxel and cisplatin). This case aims to improve the understanding of immunotherapy for advanced sinonasal malignancies and improve the treatment efficiency and survival quality of patients.

## Case presentation

A 64-year-old male presented to our hospital on May 18, 2023, with the chief complaint of nasal congestion with blood in the nasal discharge for more than 10 years. The patient was healthy and had no cancer family history. After the onset of the disease, the patient did not take it seriously and was not given medical attention. Anterior rhinoscopy showed a huge mass in the right nasal cavity, occupying the entire nasal cavity, with a poorly smooth surface and pseudomembrane. He was admitted to our hospital with “nasal mass”.

**Figure 1:**
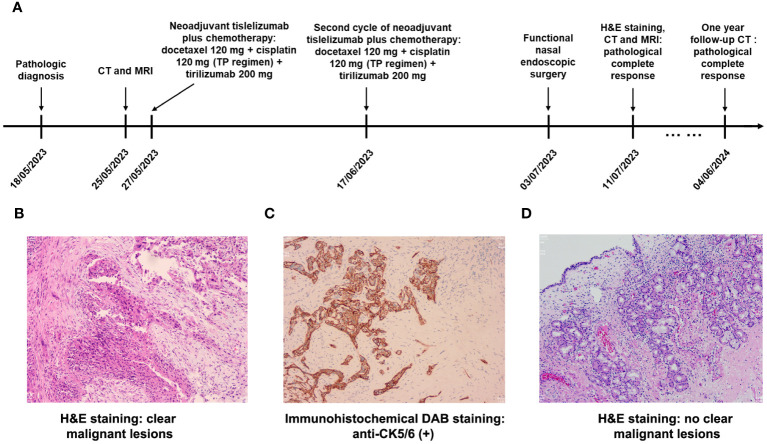
**(A)** The treatment and follow-up timeline for the patient; **(B)** Routine hematoxylin and eosin (H&E) staining (×100) revealed non-keratinizing squamous cell carcinoma at the time of diagnosis; **(C)** Immunohistochemical DAB staining (×100) showed high expression of CK5/6 on cancer cells; **(D)** H&E staining (×100) revealed chronic inflammation of the mucosa with no clear malignant lesions after neoadjuvant tislelizumab plus chemotherapy.

**Figure 1A** shows the treatment timeline for the patient. Nasal endoscopic biopsy of right inferior nasal passage confirmed that the patient was diagnosed with non-keratinizing squamous cell carcinoma (**Figure 1B**). Immunohistochemical results showed that anti-CK (+), anti-P40 (+), anti-CK5/6 (+), anti-Ki67 (+, hot spot area 80%) and anti-EGFR (++++) in cancer cells were high positive and anti-EBER (-) was negative (**Figure 1C**).

**Figure 2:**
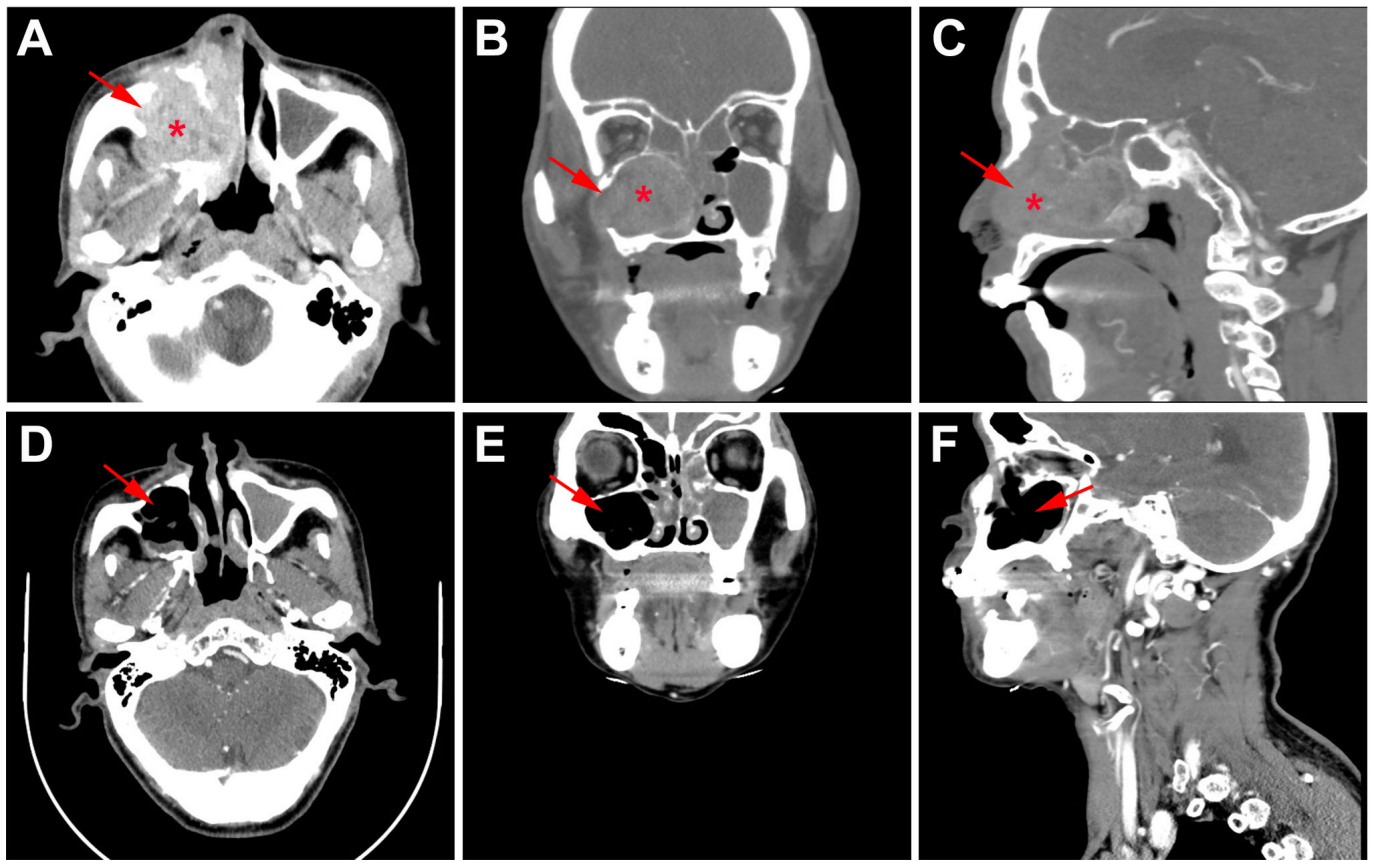
CT findings of the case. **(A)** Horizontal, **(B)** coronal, and **(C)** sagittal CT of sinus before primary treatment showed nasal tract mass accompanied by surrounding bone destruction and multiple slightly enlarged lymph nodes; **(D)** Horizontal, **(E)** coronal, and **(F)** sagittal CT of sinus after 2 cycles neoadjuvant tislelizumab plus chemotherapy showed no tumor lesions.

**Figure 3:**
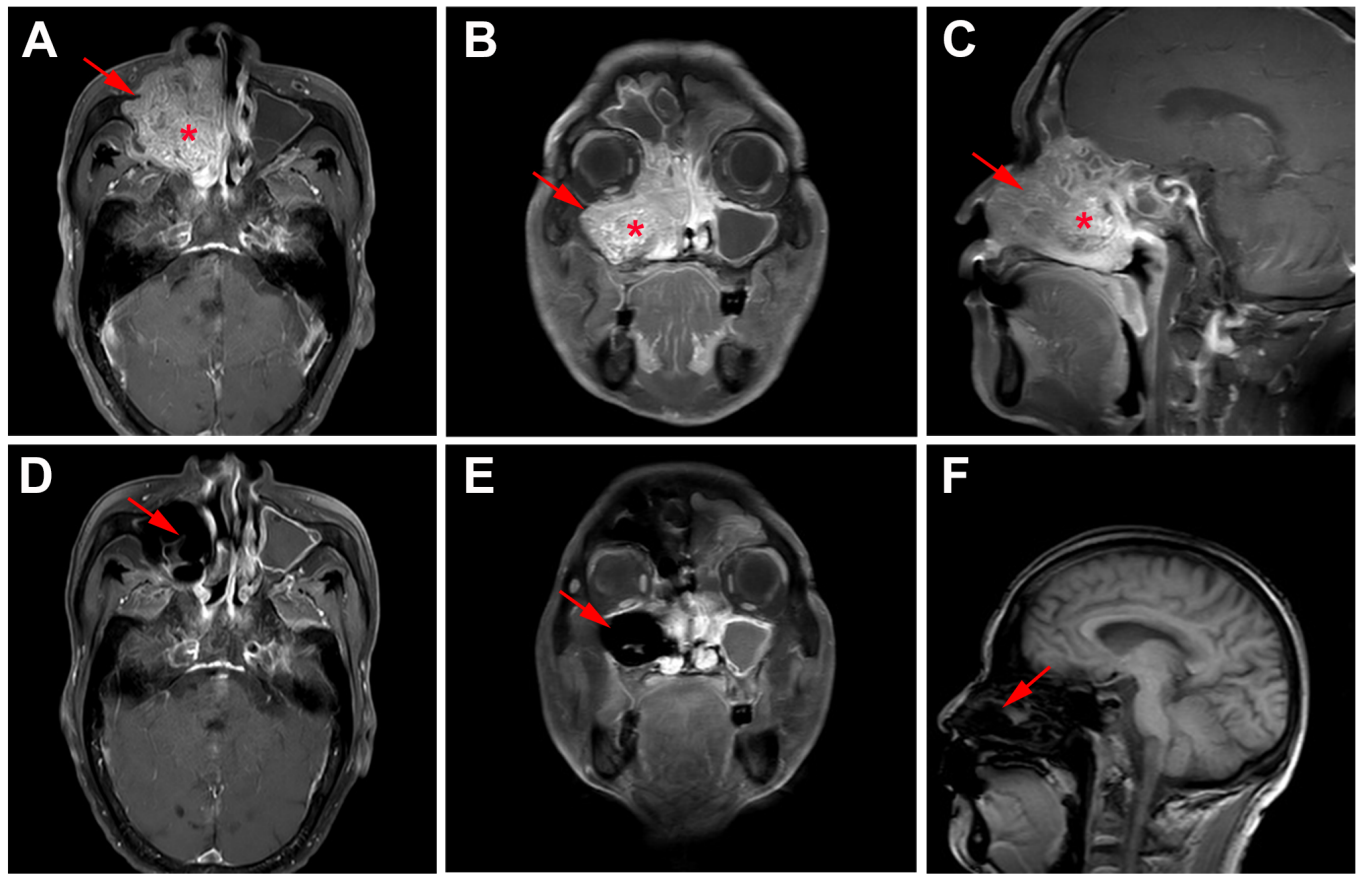
MRI findings of the case. **(A)** Horizontal, **(B)** coronal, and **(C)** sagittal MRI of sinus before primary treatment showed abnormal signal in right sieve sinus, maxillary sinus and nasal tract and mucosal thickening of the frontal sinus, the sieve sinus, the maxillary sinus, and the right pterygoid sinus; **(D)** Horizontal, **(E)** coronal, and **(F)** sagittal MRI of sinus after 2 cycle neoadjuvant tislelizumab plus chemotherapy showed no tumor lesions.

**Figure 4:**
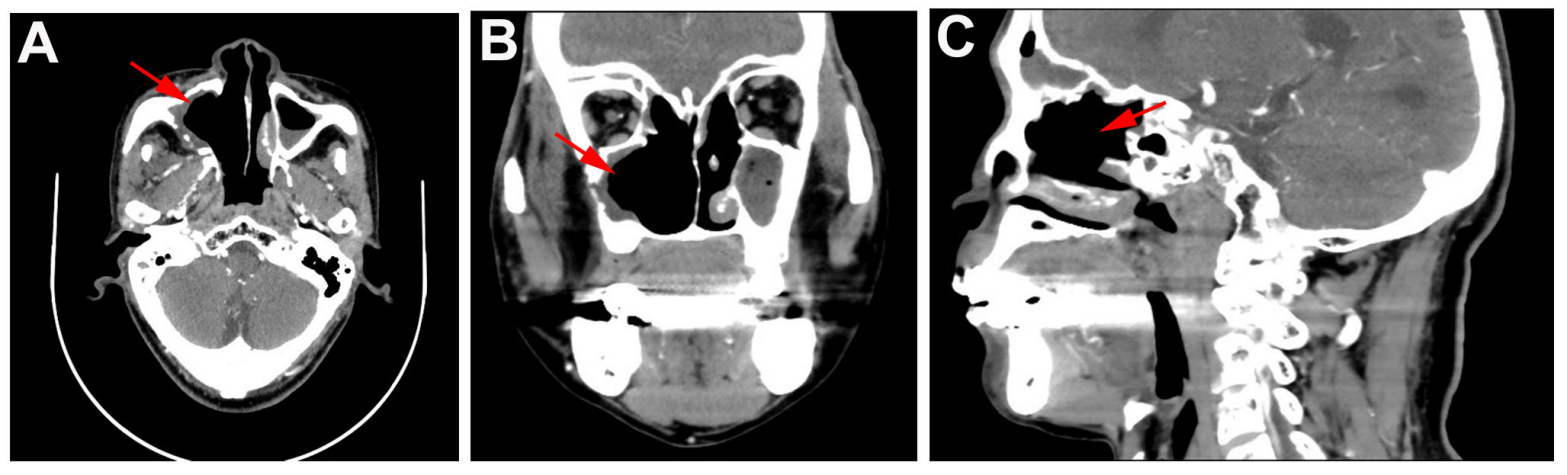
CT findings of patient in one year follow-up. **(A)** Horizontal, **(B)** coronal, and **(C)** sagittal CT of sinus in one year follow-up showed no tumor lesions.

Whole body bone scan was performed on May 25, 2023. Enhanced computed tomography (CT) of sinus revealed right maxillary sinus-nasal tract and left nasal tract mass, bilateral frontal sinus, ethmoid sinus, left maxillary sinus, and right sphenoid sinus effusion, and multiple slightly enlarged lymph nodes in bilateral carotid artery sheath area, accompanied by surrounding bone destruction, considering the possibility of sinus carcinoma. In addition, the mass grows upward and invades part of right sieve sinus (**Figures 2A–C**). Enhanced magnetic resonance imaging (MRI) of sinus showed abnormal signal in right sieve sinus, maxillary sinus and nasal tract, mucosal thickening of the frontal sinus, the sieve sinus, the maxillary sinus and the right pterygoid sinus on both sides, and hypertrophy of the left middle and inferior turbinate, considering malignant neoplastic lesions, such as sinus carcinoma, adenoid cystic carcinoma, etc., invading the right maxillary sinus bone wall, cribriform plate, and right turbinate (**Figures 3A–C**, **4B**).

On May 27, 2023, the first cycle of neoadjuvant tislelizumab plus chemotherapy was started with the following regimen: docetaxel 120mg + cisplatin 120mg (TP regimen) combined with tirilizumab immunotherapy 200mg. The second cycle of neoadjuvant tislelizumab plus chemotherapy was performed on June 17, 2023, with the same regimen: docetaxel 120mg + cisplatin 120mg (TP regimen) combined with tirilizumab immunotherapy 200mg.

Functional nasal endoscopic surgery was performed on July 03, 2023, and the resected specimen was pathologically negative, achieving pathological complete response (pCR). On July 11, 2023, H&E staining suggested: chronic inflammation of the mucosa of the right nasal sinus with polyp formation, with no clear malignant lesions (**Figure 1D**). Combined with the pathology as well as postoperative enhanced CT (**Figures 2D–F**), postoperative enhanced MRI (**Figures 3D–F**), no tumor lesions were seen, the tumor completely disappeared, and the comprehensive assessment of the tumor condition control was stable. The patient had no obvious toxic side effects of drugs and good physical status. The patient had no special discomfort and continued to receive supplemental radiotherapy to prevent recurrence.

## Outcomes and follow-up

One year follow-up CT was performed on June 04, 2024 and results showed that no tumor lesions were seen, and the comprehensive assessment of the tumor condition control was stable with no recurrence (**Figures 4A–C**). Currently, clinical follow-ups are still being performed and to date, the patient has good compliance and tolerance, and no significant adverse reactions have occurred.

## Discussion

Sinonasal squamous cell carcinoma (SNSCC) makes up 61% of all sinonasal malignancies and accounts for approximately 3% of head and neck cancers (2, 4). Sinonasal malignancies have had a relatively stable prognosis over the past decade, while other head and neck cancers have seen a significant improvement (5). SNSCC most frequently locates in the maxillary sinus and often presents with an advanced stage due to nonspecific early symptoms (6, 7). A thorough clinical examination of head and neck including all mucosal surfaces and cranial nerve examinations are required for patients with SNSCC via sinonasal endoscopic examination followed by CT and MRI. Endoscopic examination of the sinonasal area is necessary to evaluate the tumor extent and obtain tissue for histopathological examination. CT is valuable to examine the structural changes and erosion of bony landmarks, and it is essential to provide image guidance during surgery. The use of MRI can evaluate the differentiation of soft tissues regarding to invasions of the orbit, infratemporal fossa, and skull base (8).

Currently, surgical resection with negative margins is the standard treatment for resectable SNSCC, with better overall survival rates for most pathological subtypes. The importance of negative surgical margins was emphasized in a large retrospective study containing 7808 patients with SNSCC treated with different approaches (9). Several forms of multimodal treatment have been investigated for the purpose of achieving negative surgical margins, including induction chemotherapy (6, 10). If possible, salvage surgery or re-radiation therapy is the first-line treatment for locoregional recurrence; otherwise, palliative systemic chemotherapy is recommended (11). For example, palliative chemotherapy significantly improved the overall survival of patients with locally advanced sinonasal malignancies according to the report by Orlandi et al. (12). It is necessary to conduct more prospective clinical studies to determine the definite effects and benefits of adjuvant radio-chemotherapy since little data is available on adjuvant radio-chemotherapy in SNSCC.

There is still no clear definition of immunotherapy’s role in head and neck SCCs since most of these patients are excluded from clinical studies (13). However, as reported by Riobello et al. (14), cancer cells in 34% and immune cells in 45% of patients with SNSCC express membrane-bound programmed death ligand-1 (PD-L1), suggesting a potential immunotherapeutic target for SNSCC (15). A single institution retrospective analysis conducted by Park et al. (16) showed that patients with SNSCC treated with ICIs appeared to have a favorable response and trend toward improved outcomes, which is in line with HNSCC data, although no statistical significance of differences between these subgroups were obtained. Another retrospective analysis evaluated 131 SNSCCs with immunohistochemistry for PD-L1 expression, tumor-infiltrating lymphocytes, mismatch repair deficiency, EGFR alteration and HPV infection to explore antitumor efficacies of immune checkpoint inhibitors (ICIs) and the usefulness of potential predictive markers in SNSCC (17). Their results showed that 45.8% SNSCC cases presented PD-L1 expression (tumor proportion score >= 1%) and was significantly associated with worse overall survival (OS). In addition, ICIs treatment significantly improved OS of the patients with residual/recurrent EGFR-wild type tumors. In our case, anti-CK (+), anti-P40 (+), anti-CK5/6 (+), anti-Ki67 (+, hot spot area 80%) and anti-EGFR (++++) in cancer cells were high positive, and complete pathological remission was achieved after receiving ICIs treatment. The results suggest that the evaluation of immuno-markers may be helpful for selecting an individualized therapeutic strategy for patients with SNSCC.

Tirelizumab, a humanized monoclonal antibody that binds PD-1 with high affinity and specificity, can eliminate antibody-dependent phagocytosis via reducing the interaction with FcγR on macrophages (18, 19). Tirilizumab monotherapy has been shown to be effective in multiple tumors including bladder cancer (BC), esophageal squamous cell carcinoma (ESCC), gastric cancer (GC), hodgkin lymphoma (HL), hepatocellular carcinoma (HCC), nasopharyngeal carcinoma (NPC), and non-small cell lung cancer (NSCLC) (20–26). The patient reported in this case was evaluated for complete pathological remission after receiving tirilizumab monotherapy, and one year follw-up revealed that the patient is in good general condition, with no tumor progression or significant adverse effects after immunotherapy. This is the first case of SNSCC who achieved pCR after treatment with tislelizumab, which helps to encourage the inclusion of SNSCC in future prospective ICI trials.

## Conclusion

The case provides therapeutic confidence in the treatment of SNSCC, with a view to the further application of neoadjuvant tislelizumab plus chemotherapy as a treatment modality in sinonasal malignancies.

## Data availability statement

The original contributions presented in the study are included in the article/supplementary material. Further inquiries can be directed to the corresponding author.

## Ethics statement

Written informed consent was obtained from the individual(s) for the publication of any potentially identifiable images or data included in this article. Written informed consent was obtained from the patient for the publication of this case report.

## Author contributions

FC: Writing – original draft, Funding acquisition. HZ: Conceptualization, Writing – review & editing. YL: Writing – review & editing, Funding acquisition. TL: Writing – review & editing, Data curation. TZ: Data curation, Writing – review & editing, Conceptualization.
